# InSAR data reveal that the largest hydraulic fracturing-induced earthquake in Canada, to date, is a slow-slip event

**DOI:** 10.1038/s41598-022-06129-3

**Published:** 2022-02-07

**Authors:** Thomas S. Eyre, Sergey Samsonov, Wanpeng Feng, Honn Kao, David W. Eaton

**Affiliations:** 1 Department of Geoscience, University of Calgary, Calgary, Canada; 2 Canada Centre for Mapping and Earth Observation, Natural Resources Canada, Ottawa, Canada; 3 Guangdong Provincial Key Laboratory of Geodynamics and Geohazards, School of Earth Sciences and Engineering, Sun Yat‐sen University, Zhuhai, 519082 China; 4 Southern Marine Science and Engineering Guangdong Laboratory (Zhuhai), Zhuhai, 519082 China; 5 Geological Survey of Canada, Sidney, Canada; 6 University of Victoria, Victoria, Canada

**Keywords:** Seismology, Geophysics, Tectonics

## Abstract

For tectonic earthquakes, slip rate spans a continuum from creep to supershear earthquakes, where slow slip events (SSEs) are important in releasing stress without radiating damaging seismic energy. Industrial-scale subsurface fluid injection has caused induced earthquakes, but the role of SSEs in fault activation is currently unclear. Ground-deformation observations, measured by satellite radar, show that SSEs up to magnitude 5.0 occurred during hydraulic fracturing (HF) operations in northwestern Canada, corroborated by reported deformation of the steel well casing. Although the magnitude 5.0 SSE exceeded the magnitude of the largest induced earthquake in this region (magnitude 4.55), it was undetected by seismograph networks. The observed SSEs occurred within a buried thrust belt and their magnitude and duration are consistent with scaling behavior of SSEs in unbounded natural systems, e.g. slab interfaces in subduction zones.

## Introduction

Many natural fault systems exhibit a continuum of behavior, from dynamic rupture (conventional earthquakes) to slow (aseismic) slip^1,2^. With durations that range from days to years^3^, SSEs have been documented in most subduction zones^2–6^ and have also been observed in other tectonic environments such as strike-slip faults^2,7,8^. In subduction zones, deep SSEs occur at the transition from brittle to ductile deformation (20–50 km depth) and are thought to be related to the frictional rheology and temperature-driven dehydration of the subducting slab, which increases the pore pressure^9^. Slow slip plays an important role throughout the earthquake cycle, as it releases stress aseismically in some fault regions. However, the aseismic slip can increase stress in unstable zones, leading to dynamic rupture^10^. Improved understanding of the dynamics of slow slip can therefore aid in more accurate earthquake hazard characterizations^1^.

A causal link is well established between fluid injection and seismic slip during anthropogenic (induced) earthquakes^11^. Injection can also induce aseismic slip, similarly to natural fault systems; direct evidence for such behavior comes from laboratory measurements^12,13^ and small scale in-situ experiments^14,15^. Borehole deformation believed to result from predominantly aseismic slip during fluid injection has also been observed^16–18^. Indirect evidence of aseismic slip is furnished by microseismicity migration patterns^16^, but slip quantification is difficult. Some studies found possible evidence of long-duration slow earthquakes with tremor-like waveforms^19,20^; however, many of these observations have since been refuted as misinterpreted regional earthquakes^21,22^, or even due to anthropogenic sources^23^. Geodetic data shows that shallow aseismic slip within the Brawley geothermal field, USA, triggered activity in a deeper seismic swarm^24^. Here, we present satellite observations, independently corroborated by reports of well casing deformation during operations, that reveal multiple SSEs during hydraulic fracturing.

## Results

**Figure 1:**
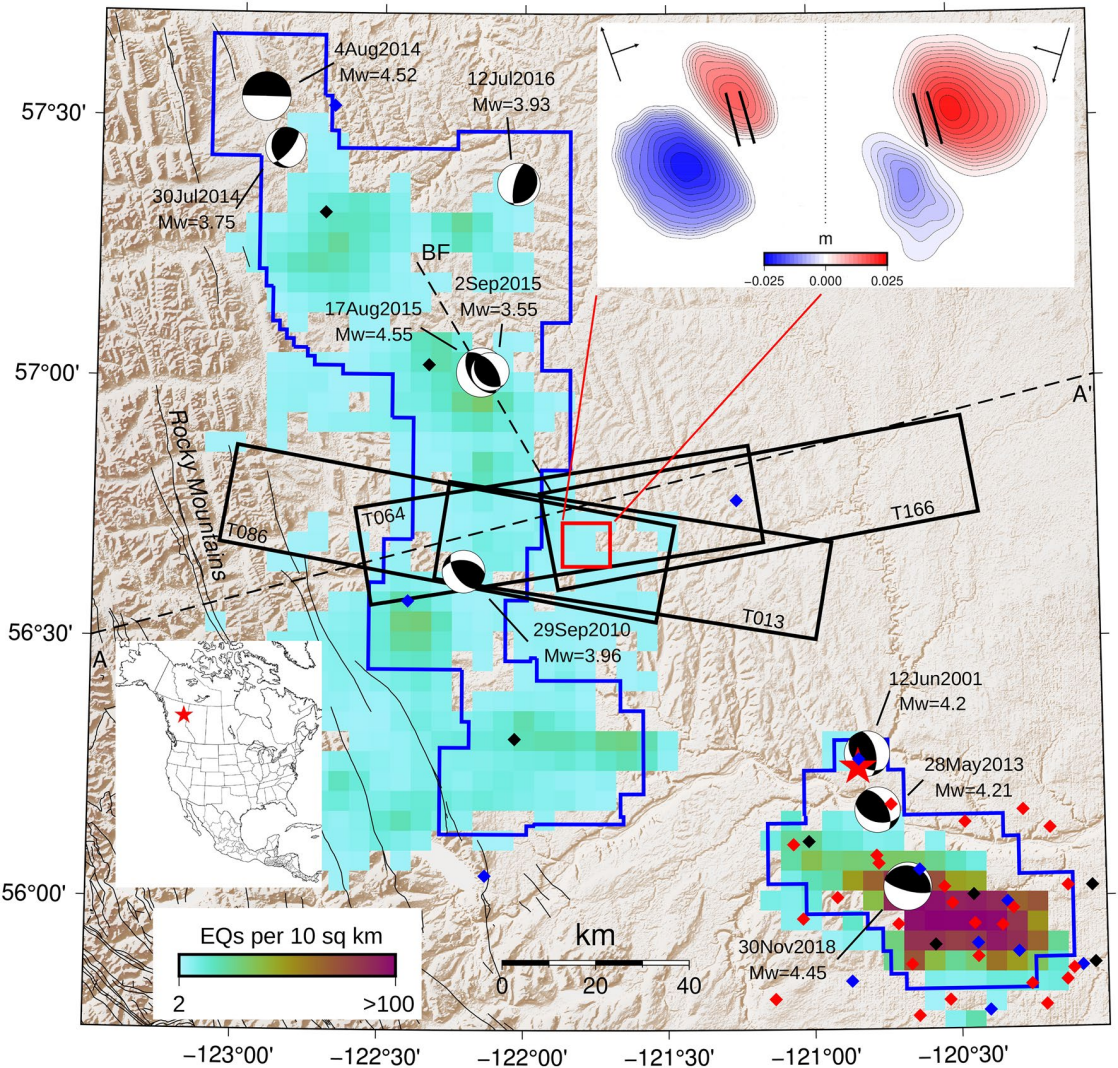
Map of the study area and regional seismicity in NE British Columbia. The study area is shown as a red square. Ground displacement for September 2017 (inset, upper right) is shown for tracks 64 (left) and 13 (right). Satellite track images used in this study are shown as labelled black rectangles; large and small arrows denote azimuth and line-of-sight, respectively. Spatial density of seismicity is indicated by the colorscale; largest events are labelled with focal mechanisms. Seismic stations (diamonds) are color-coded (blue—installed before first event, black—installed after first event but before second event, red-installed after second event). The town of Fort St. John is shown (red star). Dashed line A–A’ shows location of the cross-section Fig. 4; *BF* Blueberry Fault. Created using^86,87^ (see “Acknowledgements” for full details).

**Figure 2:**
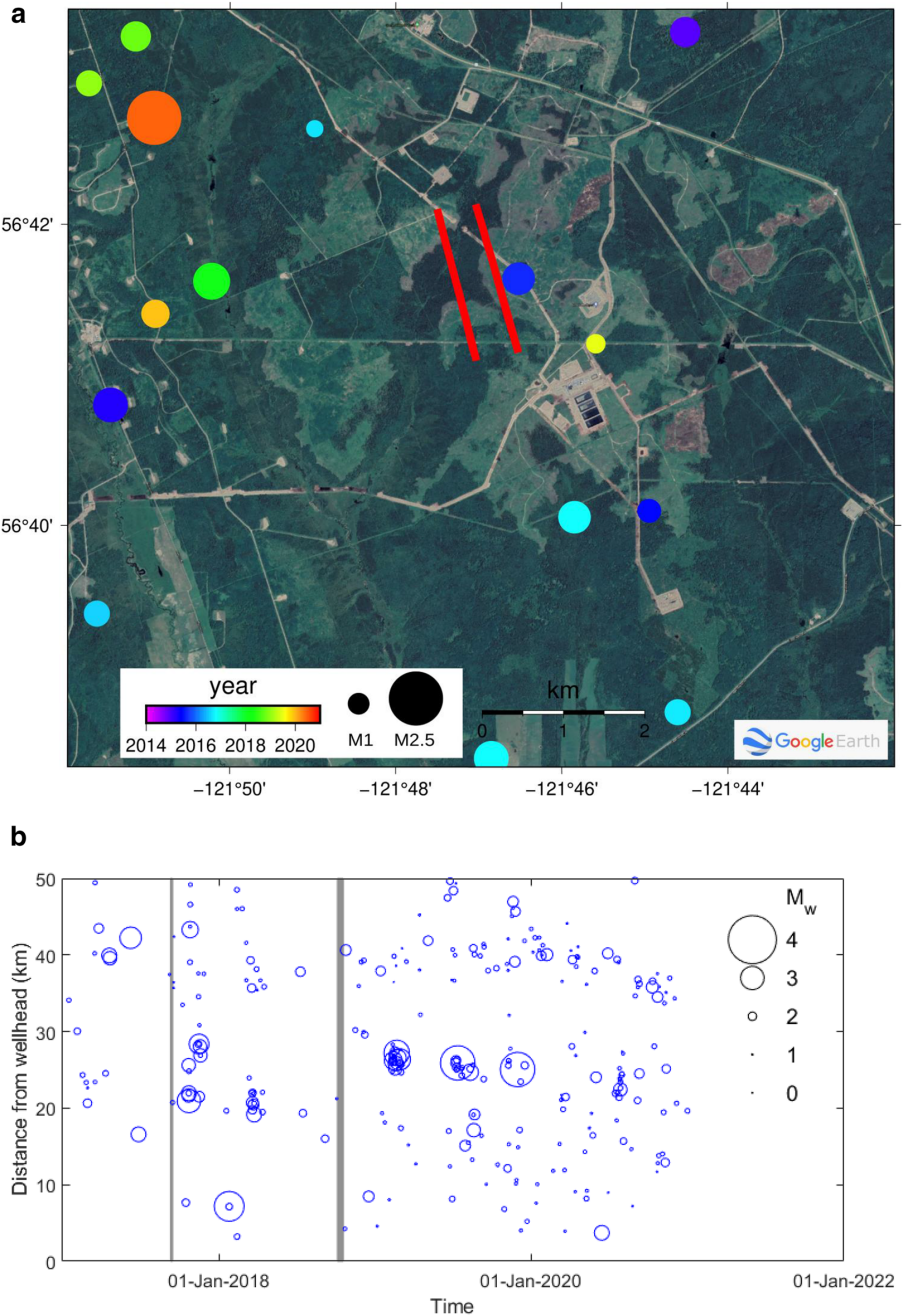
Seismicity in the region recorded on the regional network. **(a)** Map of events during the period 2014–2021 colored by time and scaled by magnitude. No major events were recorded, including during the slip episodes in 2017 and 2018. Horizontal wells are shown in red. **(b)** Seismicity over the time period 2017–2020 plotted versus distance to the wellpad and scaled by magnitude. Times shaded in grey correspond to the approximate times at which the SSEs occurred; there appears to be no spatiotemporal correlation with significant seismic events. Created using^86,87^ (see “Acknowledgements” for full details).

Interferometric synthetic-aperture radar (InSAR) is a technique for mapping ground deformation using radar images collected at different times. We obtained satellite InSAR data for a region NW of Fort St. John, British Columbia, Canada, in the foreland of the Rocky Mountains (Fig. 1). The data reveal consistent patterns of paired uplift and subsidence that took place in the same region in September 2017 and October 2018, respectively (Supplementary Figs. 1 and 2). No other deformation signals are distinguished in the Sentinel-1 data for this region from 2015 to present. The observed patterns of ground deformation are indicative of shear dislocations on faults^25^. The models that best fit the data and regional setting are shallowly-dipping (8°) thrust events with magnitudes of *M*_*w*_ 5.0 and *M*_*w*_ 4.2 and depths of 1.8 km and 1.7 km, respectively (“Methods”; Supplementary Figs. 3 and 4). The regional seismic network did not detect any earthquakes in this region during the two time periods (Fig. 2; Supplementary Table 1), despite a network detection threshold in this region of ~ *M*1.5 (“Methods”). The InSAR deformation signals, together with the absence of detected seismicity, are best explained if these are SSEs.

Induced seismicity is known to occur in this region, primarily due to HF of the Montney Formation (a relatively thick (> 200 m), fine-grained siltstone^26^), with a record of induced events up to *M*4.55 to-date^26^. HF is the process of injecting pressurized fluids into low-permeability hydrocarbon reservoirs in order to create open fracture systems to increase permeability and achieve economic flow rates^27^. To maximize efficiency, horizontal wellbores are drilled into reservoir formations for distances up to several kilometers. HF is completed in multiple stages along the wellbore, from the distal end of the horizontal well (toe) to the proximal end (heel).

**Figure 3:**
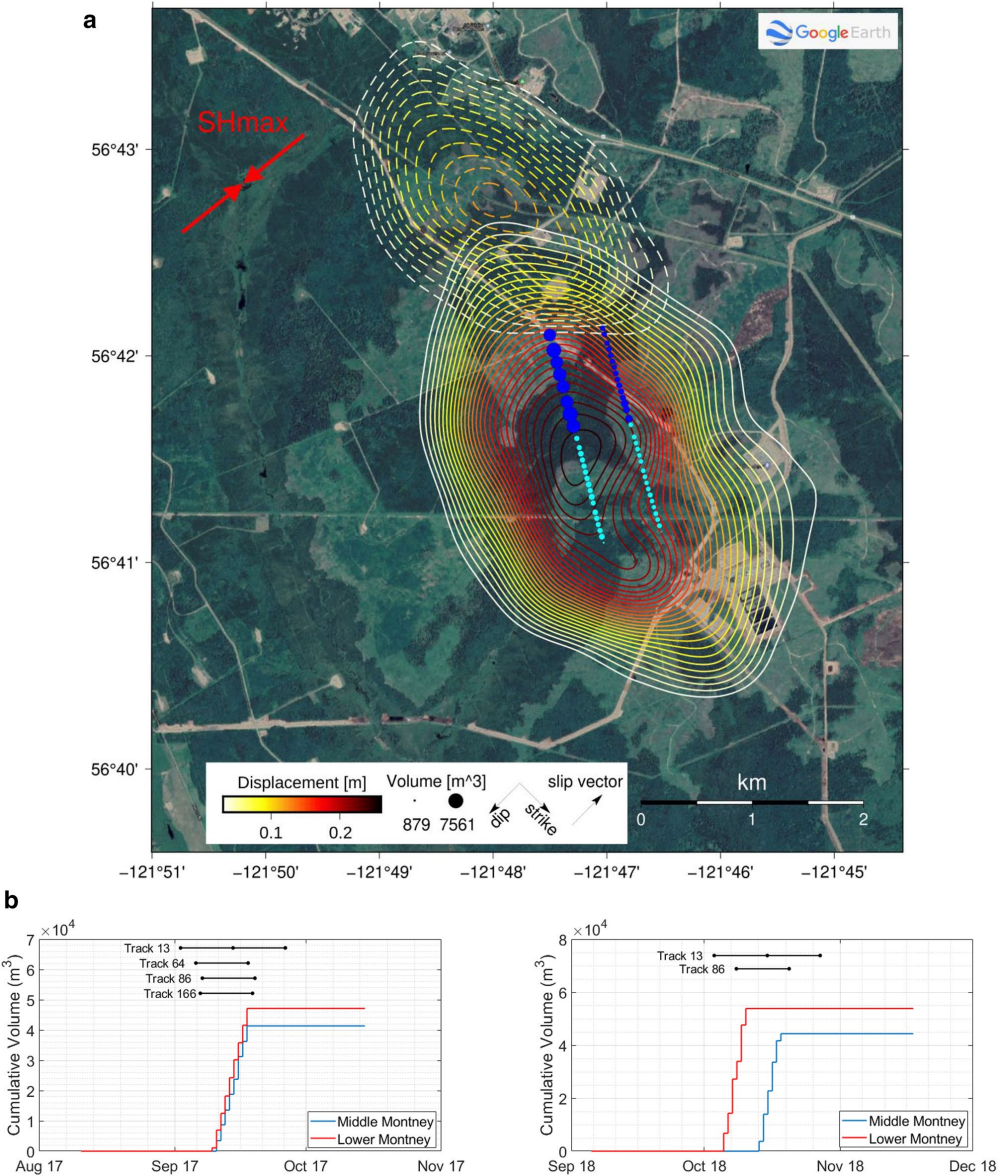
Modelled slow slip events and spatiotemporal relationship to hydraulic fracturing. **(A)** Location of modelled slip; contours show slip (2017 event—solid lines; 2018 event—dashed lines). Hydraulic fracturing stages are shown as circles, scaled to volume injected (cyan = 2017, blue = 2018). Strike, dip, slip vector and *S*_*Hmax*_ are labelled. **(B)** Cumulative fluid volume injected for the Middle and Lower Montney wells and InSAR data acquisition times (black circles) for 2017 (L) and 2018 (R) spanning periods of measurable deformation used to help constrain SSE timing. Created using^86,87^ (see “Acknowledgements” for full details).

At the time of the InSAR-detected slip, two horizontal HF wells were injecting at ~ 2 km depth (Fig. 3a). The injection operations at these two HF wells were unusual. Typically, hydraulic fracturing treatments are completed over a short time scale (usually days or weeks) prior to putting the wells onto production. However, public records submitted by the operator show that HF was split into two time periods in 2017 and 2018, with half of the planned program completed in 2017 (Supplementary Table 4). The two time periods of fluid injection each bracket the timing of the identified InSAR events (Fig. 3b). In addition to proximity in location and depth, the slip also migrates northwards (Supplementary Fig. 5), consistent with staging of the HF operations.

The reason for the unusual one-year delay in the HF program is evident from the completions report submitted by the operator, which shows that deformation of the steel casing in each well was recorded in both 2017 and 2018 (“Methods”). This led to the cessation of operations in 2017 when some of the equipment used to conduct HF became stuck in the wellbore. These observations are consistent with the InSAR-derived model of shallowly-dipping slip immediately above the horizontal wellbore sections, as the modelled slip patterns for 2017 and 2018 both imply sufficient slip at the wellbore locations to induce casing deformation (Fig. 3). Although the reports for these wells do not specify the amount of deformation, elsewhere, HF of the Montney Formation has caused wellbore casing deformation marked by bedding-plane slip of up to 50.4 mm without any observed accompanying seismicity^17,18^ (“Methods”). The observations of wellbore deformation further corroborate the slip locations, timing and connection to the HF operations.

## Discussion

**Figure 4:**
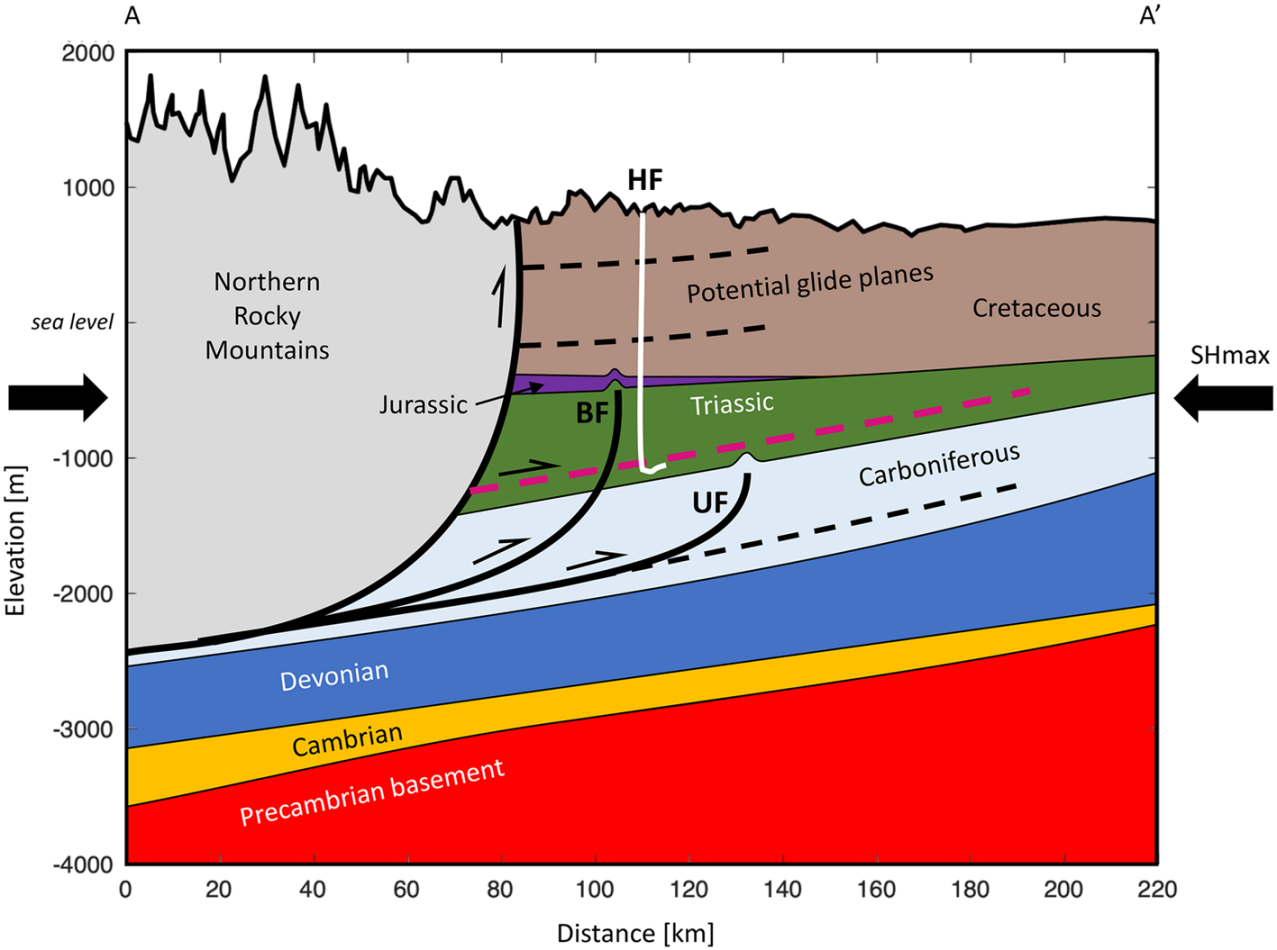
Schematic interpretation. Cross-section showing the structure of the Rocky Mountain foreland thrust belt in the region, based on^54^. Glide planes form in weaker and/or overpressured sedimentary layers^28^; the inferred glide plane for the SSEs is shown in magenta. *HF*  hydraulic fracturing wells, *BF*  Blueberry Fault, *UF*  unnamed fault; ~ 28 × vertical exaggeration.

The shallow dip of the slip plane does not align with the estimated dip of mapped thrust faults in this region (“Methods”). However, the thin-skinned thrust belt is characterized by ramp-flat geometry, and the slip episodes occurred along shallowly-dipping bedding (glide) planes primarily within the Montney Formation (Fig. 4). Slip along regional glide surfaces is documented within the Rocky Mountain foothills^28,29^ and occurs along weak boundaries between sedimentary layers. It also occurs preferentially in naturally overpressured formations^29,30^, which supports our model since the Montney Formation is overpressured in this region^31^. During high pressure injection, slip along bedding planes can be caused by localized horizontal shear on weak planes at the top of injection intervals due to the reduction in normal stress and reservoir expansion^32^. In addition, evidence for bedding-plane slip is commonly observed in microseismic surveys during HF^33^. In extreme cases where the injection pressure exceeds the vertical principal stress, tensile opening (hydraulic jacking) of the surface may occur prior to shear displacement^32^. In such cases, the expected slip direction is parallel to the direction of maximum horizontal stress (*S*_*Hmax*_)^18^, in good agreement with the model (Fig. 3), as *S*_*Hmax*_ in this region is oriented N45°–55°^31,34^. Numerical simulation of the slip mechanism is beyond the scope of this observational study, but given the close proximity of the glide plane above the HF injection stages, it is likely that induced pore pressure and poroelastic stress changes on the plane were significant.

**Figure 5:**
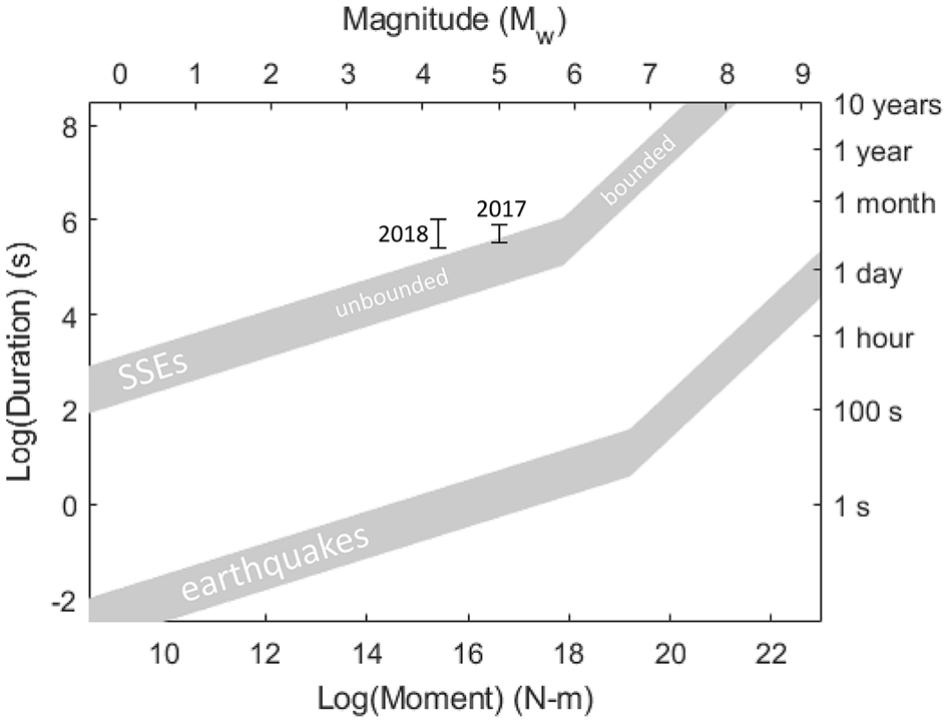
Slip event scaling and comparison to typical scaling relationships. Scalar moment (*M*_*0*_) versus duration for the two observed slip events. *M*_*0*_ is well constrained but duration is plotted as a possible range, assuming each is a single distinct event. The events plot close to the region that describes (unbounded^6^) SSE scaling for the Japan and Cascadia subduction zones^6,35^.

In natural settings, SSEs are distinguished from earthquakes by their orders-of-magnitude lower slip rates, propagation velocities and stress drops, and much longer durations^1^. A scaling relation between event magnitude (or seismic moment, *M*_*0*_) and duration (*t*) has been proposed where ${M}_{0}\propto {t}^{n}$. Recent studies have suggested that ${M}_{0}\propto {t}^{3}$, similarly to regular earthquakes^35^. It has also been proposed that a transition in scaling between *n* = 3 and *n* = 1 occurs where the slip patches are large enough to reach the up- and down-dip boundaries of their slip zones, i.e. where the slip regime changes from unbounded to bounded^6^. Nevertheless as shown in Fig. 5, the events appear to plot close to those observed in natural settings^6,35^, and perhaps show similar *n* = 3 scaling, although from two data points this is inconclusive (“Methods”). Due to the stratified nature of sedimentary basins, the bedding surfaces can be considered as unbounded.

It is curious that such significant slip events did not result in detectable seismic events (earthquakes). Well-located induced seismic events within the immediate region generally occur within the massive carbonate Debolt Formation that underlies the Montney^34^. In this region, this suggests that the Montney Formation is too weak to sustain larger seismic events, especially as stress changes due to injection are higher within the Montney than the Debolt Formation. Events with low slip velocity have been hypothesized to occur in regions of low effective stress^36^, with low shear wave velocity^37^, and/or with various rock properties such as high total organic carbon and clay content^10,30,38^. These conditions may apply to the Montney Formation due to the shallow location, formation overpressure^31^, high pressure fluid injection and rock composition. Similar to decoupling of deformation by décollements in thin-skinned thrust belts such as the Canadian Rockies, the shallow dip of the events may mean that slip-induced stress changes are insufficient to generate significant seismicity in deeper seismogenic layers^10^.

Our observations are in agreement with evidence that a large fraction of the energy budget for HF operations is released by aseismic deformation processes^39^. Since SSEs during HF operations have not been previously documented, this investigation may have captured particularly large events (“Methods”) that lie just above the noise floor for InSAR data. The shallow depth of this event (< 2 km) also made it more easily detectable using InSAR than an equivalent magnitude event at greater depth. Increasing recognition of casing deformation during hydraulic fracturing^17^ could mean that aseismic slip such as this is not uncommon.

Our study suggests that thrust belts activated by fluid injection can exhibit a range of slip behavior analogous to subduction systems, ranging from SSEs to dynamic rupture. Indeed, both of these systems are characterized by shallow-dipping interfaces with slip dynamics controlled by frictional rheology and fluctuations in pore pressure^9^. Use of InSAR to monitor fast and slow deformation processes in areas of fluid injection promises to yield new insights into links between SSEs, casing deformation and earthquakes in this setting.

## Methods

### InSAR data and modelling

In this study, we used Sentinel-1 SAR data from two ascending (064 and 166) and two descending (013 and 086) tracks (Supplementary Table 2). Sentinel-1 data is of the best quality and coverage for this region and time period, and is the only available SAR data for the main slip event from the main SAR satellites, e.g. RADARSAT-2. The interferometric wide (IW) single-look complex (SLC) images with 2.3/14.9 m range/azimuth spatial resolution were downloaded from the NASA Distributed Active Archive Center (DAAC) operated by the Alaska Satellite Facility (ASF). The individual bursts covering the AOI were extracted and processed using GAMMA software^40^. Interferograms were computed using 16/4 range/azimuth multilooking and the topographic phase was removed using the 30 m resolution ASTER DEM. Differential interferograms were filtered using adaptive filtering with a filtering function based on the local fringe spectrum^41^, unwrapped using the minimum cost flow algorithm^42^, converted to the line-of-sight displacements, and geocoded (Supplementary Figs. 1, 2, 5). SAR images and interferograms used in this study are shown in Supplementary Tables 2 and 3.

Using the injected fluid volume data (Supplementary Table 4), we first investigated whether the observed ground deformation could be caused purely by the volume of injected fluid. The upper limit on deformation is estimated assuming a pure elastic instantaneous response due to a Mogi source^43^. This shows that the maximum ground uplift due to injection is ~ 5 mm in 2017 if the entire volume was injected at once, which corresponds to maximum line-of-sight measurements for both Track 013 and Track 064 of ~ 4 mm (Supplementary Fig. 6), much lower than the observed displacements. In addition, the displacement patterns are very different to those observed. Consequently, an inflation source cannot explain the observations and thus the observed patterns of combined uplift and subsidence are better explained by shear dislocations on faults^25^. Other sources of deformation, e.g. landslides, can be ruled out because the signal does not correlate with the topography.

Only high-quality interferograms (Supplementary Table 3) were used in the modeling, while others were used for qualitative confirmation of the deformation signal. For the 2017 period, we used one ascending and one descending interferogram. This data allowed us to solve for distributed slip on a fault of constant strike, dip, and rake (Supplementary Fig. 3). For the 2018 period, we used the previously-derived fault geometry and solved for distributed slip using the only available two descending interferograms.

To account for the InSAR surface deformation observed in the two periods of 2017 and 2018, we conducted slip modelling using a geodetic inversion package, PSOKINV^44,45^. This package is tailored for geodetic data modelling, particularly using InSAR observations including a novel nonlinear optimization, modified particle swarm optimization (MPSO), to conduct global optimal parameter searching with limited human intervention, based on a half-space elastic model^44,46^. In this study, we applied a two-step inversion strategy, in which we first performed a nonlinear global search for the fault geometric parameters based on the accumulated InSAR surface deformation of the 2017 event and then carried out distributed slip inversions with the fixed fault geometries determined in the previous step for the two periods, respectively. The nonlinear inversion result shows that a shallowly dipping thrust fault having a strike of 136° and dip of 8° could be responsible for the surface deformation with an optimal rake angle of 94°. This model gives very low residuals for each track and in both 2017 and 2018 (Supplementary Fig. 4). Parameter sensitivities are shown in Supplementary Fig. 7. The maximum slip of 0.24 m for the 2017 event is concentrated at depth of ~ 1.8 km relative to the local surface, while the maximum slip of 0.11 m for the 2018 event is revealed at a depth of ~ 1.7 km (Supplementary Fig. 3). The geodetic moments are 4.24 × 10^16^ Nm and 2.09 × 10^16^ Nm, corresponding to moment magnitudes (*M*_*w*_) of 5.0 and 4.2 for the two events, respectively.

It was found that a normal fault with a strike of 144° and a dip of 41° also appears to fit the InSAR data equally well given the non-unique nature of the inversion, with an optimal rake of −108°. We discarded this model early on as it does not fit with the regional stress regime (“Regional stress regime” section), and it is highly unlikely that faults would be reactivated in an opposite sense to that expected from the regional stress field. This interpretation is supported by focal mechanisms for induced earthquakes in the region, which show a thrust mechanism (Fig. 1).

### Geological setting

The Triassic Montney Formation is a relatively thick (> 200 m), fine-grained siltstone with low matrix permeability of 10^–8^–10^–7^ µm^2^^47^. It is typically subdivided into three Members: the Lower, Middle and Upper Montney Members^48^. The Montney is a “stacked play”, wherein multiple horizontal wells are often drilled from a single wellpad into different depth levels.

The Montney Formation is underlain by the Permian Belloy Formation, comprised of interbedded siliciclastics and carbonates which is < 10 m thick in this region. Below this are thick (~ 500 m) massive carbonates of the Carboniferous Rundle Group, the topmost of which is the Debolt Formation. These formations in turn overlie the Banff Formation, a thick shale sequence of Late Devonian age.

To the west of the study region, the Rocky Mountains are a classic example of a foreland thrust belt characterized by thin-skinned tectonics, such that there is no significant involvement of the Precambrian crystalline basement^49^. In the final stages of this orogen (Late Cretaceous–Paleocene), tectonic shortening occurred in the foreland through fault-propagation folds underlain by blind thrusts^34,50^. 3D seismic data from the region shows clear thrust ramps separated by ~ 2 km^34^. The faults strike NNW-SSE, parallel to the Rocky Mountain deformation front to the west, and dip to the SW at ~ 30°–40° at the depth of the reservoir. One significant thrust fault is the Blueberry Fault, which is located in the west of the study region^51^. In this thrust system, faults flatten out and sole into the Banff Formation, forming a basal décollement zone throughout the region^52^. A number of strike-slip faults have also been mapped, from the Precambrian basement^53^ to shallower units within the sedimentary section^34^.

The geological cross section shown in Fig. 4 was constructed using map information available in^54^. First, a surface topography profile was produced using topographic information from Google Earth between the points 56.5 N 123.5 W and 57.0 N 120.0 W, which is approximately perpendicular to the local strike of faults within the Canadian Rocky Mountains at this latitude. The depth of the top of Precambrian basement below mean sea level was digitized along this profile, based on contour data in Fig. 5.1 of^55^. Above this, layers representing the Cambrian, Devonian, Carboniferous, Triassic, Jurassic and Cretaceous systems were digitized and drawn in the section based on isopach maps from the following sources:

- Cambrian:^56^
- Devonian: Summation of isopach data for the Elk Point Formation^57^, Beaverhill Lake Group^58^, Woodbend and Winterburn Groups^59^ and Wabamun Formation^60^.
- Carboniferous:^61^
- Triassic:^62^
- Jurassic:^63^
- Cretaceous:^64^

Note that Permian strata are present along the profile but they are too thin to be shown in the cross section at this scale. The cross section was validated by comparing the depth to the pre-Cretaceous unconformity determined using this approach with structure contours from^64^.

Seismicity within the region has increased significantly since development of the Montney unconventional resource play began, in 2005^65,66^. Induced seismicity has primarily been attributed to hydraulic fracturing^67^, although a small number (two of 104 active wells reported in 2014) of saltwater disposal wells also induced earthquake activity^65^. The largest induced earthquakes were a *M*_*w*_ 4.55 earthquake in 2015, to the north^68^, and a *M*_*w*_ 4.45 earthquake in 2018^69^ to the south of this study area (Fig. 1). There is evidence that hydraulic fracturing within the Lower Montney Member is more seismogenic than injection into shallower levels^65^, and the largest events were associated with Lower Montney completions^68,69^. One of the two wells that were undergoing HF when the SSEs occurred targets the Lower Montney.

### Earthquake monitoring

There has been a concerted effort since 2013 to densify the seismograph network in western Canada for induced earthquake monitoring^66,70^. In addition to many local seismic arrays established by the private sector, the number of seismograph stations established with public funds has increased from 21 in 2013 to 74 in 2020^71–73^. As a significant portion of hydraulic fracturing operations in northeast BC target the Montney formation in the Fort St. John–Dawson Creek area, the majority of newly established stations are located more than 60 km south of our study area (Fig. 1). Overall, the magnitude detection threshold for local seismicity varies across the region from *M*2.6 to *M*1.0 depending on the number of available stations^66^. Specifically for our study area, we estimate that the magnitude of completeness is ~ *M*1.5 based on a detailed performance analysis of the regional network^74^.

On a regional scale, the majority of injection-induced earthquakes (IIE) are observed along a 150 km wide NW–SE band of moderate strain rate in the easternmost Cordillera and foothills^75^. The distribution, however, is heterogenous with many earthquake clusters in the vicinity of a small number of wells (Supplementary Fig. 8)^26,76^. The highest IIE activity is observed in the Fort St. John–Dawson Creek area, with hundreds of events per 10 square kilometers since 2013, partly because of the higher station density. In contrast, the IIE density in the northern Montney drops by at least one order. Among the > 25,000 earthquakes detected and reported by the Induced Seismicity Research Project of the Natural Resources Canada^71,72,77^, only 21 events with magnitude ranging 0.8–2.5 occurred in our study area (Fig. 2, Supplementary Table 1). Significant earthquakes with *M* ≥ 3.5 are all located more than 30 km from our study area (Fig. 1).

We examined data from the closest stations to determine if any low frequency tremor signals were apparent, as is possible for slow earthquakes. In particular, the recent discovery of earthquakes characterized by hybrid-frequency waveforms (EHWs) during a hydraulic fracturing stimulation in NE BC^78^ is encouraging. These EHW events generally have lower stress drop and/or slower rupture speed that manifest the transition from aseismic slip to seismic failure. However, EHW signals are observable only at close distance (< 5 km) to the injection well. Furthermore, tremor and EHW identification would require similar waveform signals to be observed on several stations^79^. Unfortunately, given that the closest station is > 30 km away from the SSE locations and that the network is relatively sparse in this region, it is not possible to distinguish any signals that resemble previously reported tremor signals or EHWs.

### Regional stress regime

Stress data for the region is indicative of a thrust to strike-slip regime^80^. Detailed studies in the wider region have also been carried out; for example, in the Farrell Creek Field of the Montney play, ~ 50 km south of the study area^18,81^. Minimum horizontal stress (*S*_*hmin*_) gradients of ~ 21.1 kPa/m, vertical stress (*S*_*v*_) gradients of ~ 25.3 kPa/m and maximum horizontal stress (*S*_*Hmax*_) gradients of ~ 28 kPa/m were estimated, supporting a strike slip fault regime. A study^31^ conducted in the wider region encompassing the study area documented consistent results, with *S*_*hmin*_ gradients of 16.5–22 kPa/m, *S*_*v*_ gradients of 24.1–25.2 kPa/m and *S*_*Hmax*_ gradients of 26.4–32.8 kPa/m. *S*_*Hmax*_ orientations in this region are approximately N45°–55°^31,34,80^.

### Wellbore deformation due to bedding-plane slip

Bedding/glide-plane slip has been previously documented in the Montney play, manifested as wellbore casing deformation. Such slip may occur preferentially to other failure where bedding planes are not parallel to a principal stress axis, as bedding planes can be very weak. For example, from triaxial experiments the cohesion (*C*) and coefficient of friction (*μ*) of bedding plane surfaces within the Montney in the Farrell Creek Field ~ 50 km to the south were shown to be significantly less than those of the intact rock^81^: bedding planes had averages of *C* = 1.5 MPa and *μ* = 0.51, compared to *C* = 21.1 MPa and *μ* = 1.05 for intact rock. Wellbore casing deformation of as much as 50.4 mm was documented on bedding planes within the Upper and Middle Montney in the Farrell Creek Field^18^. Bedding-plane slip also caused crimping (wellbore bending) ranging from 0.1 to 0.5 mm in the Altares Field of the Montney, ~ 35 km SSW of the study area^17^. This is despite two reports that stated that no loss of integrity or impact on the vertical portions of wellbores was reported as of 2014^65^ or 2019^82^, although these reports mentioned several instances of casing deformation that are known to have occurred within the horizontal portion of shale gas wellbores targeting the Montney.

Operations for the two wells in this study were unusual in that they were completed over two distinct time periods (Supplementary Table 4), whereas typically HF treatments are completed over a short time scale (usually days) prior to initiation of production. We therefore investigated the cause of this from the operations descriptions in the public well database. The public well database reports that operations on the Lower Montney well ceased on 18 September on the Lower Montney well, due to wellbore casing problems that caused the tools used to conduct the hydraulic fracturing to become stuck in the wellbore. A caliper log was run between 2190.40 mKB to 1845.0 mKB and identified two main casing shifts, from 2077.5 to 2081.0 mKB and 2090.0–2093.0 mKB, which corresponds to 2009–2012 mTVD and 2016–2018 mTVD below surface. Unfortunately, no data are provided on the magnitudes of these deformations, but for the tools to become stuck we infer that this deformation was substantial, i.e. cms: for reference, the wellbore diameter at this depth was 139.7 mm. A tight spot in the well casing affecting operations was also reported while retrieving downhole tools after operations (completion of Stage 25) in 2018 at 2083 mKB. These zones are located within the Middle Montney Member. Well logs and geological interpretations are provided in the public database, but there is no obvious difference between the geological interpretation and well log data for this zone than the surrounding zones that suggests a specific zone of weakness. Geological interpretation derived from wellbore cuttings for this well throughout this section of the Middle Montney Member describes medium to dark grey shaly siltstone with light grey silty shale stringers. A similar lack of correlation was shown between casing deformations within the Middle and Upper Montney and lithofacies for multiple wellbores in the Farrell Creek region^18^. Another possibility is that this zone corresponds to the Altares Member, which contains higher carbonate content and is hypothesized to affect wellbore deformation in the nearby Altares field due to frequent changes in geomechanical properties between the interbedded siltstone, bioclastic packstone and grainstone beds which provide numerous planes of weakness^17^. Bedding-plane slickensides are common and reflect lateral movement at these lithofacies contacts. However, it is unclear whether the Altares Member extends through the study area as it is relatively poorly documented.

Similar observations are also made for the Middle Montney well. Operations ceased on 19th September 2017 due to casing deformation. The main deformation that affected the downhole tools was located at 2177.6 mKB (1990.5 mTVD), but multiple deflections of 1 mm were reported at the casing collars at 2116.20 mKB, 1970.40 mKB, 1956.40 mKB, 1914.10 mKB, 1899.40 mKB, 1870.60 mKB, 1813.90 mKB and 17114.0 mKB. Another deformation event was recorded during resumed operations in 2018 at 1840 mKB (1802 mTVD), approximately at the interface between the Doig siltstone and Halfway sandstone units. These observations suggest a thick shear zone of multiple weak planes rather than a single detachment surface.

### Time constraints on SSEs

We estimated the minimum and maximum time periods over which the majority of slip in the SSEs occurred. The maximum time periods are given as the period from the final InSAR image showing no deformation or time of first injection (whichever is later), to the first InSAR image showing the full deformation pattern. The minimum time periods assume a single slip event and are given as the final termination of injection (as this was affected by borehole deformation related to the slip) minus the earliest InSAR image showing deformation. It is noted that due to the discrete time sampling we cannot rule out an episodic slip nature, which may be possible due to the periodic nature of the HF injection and distinct stage locations.

For 2017, the minimum time period of slip is therefore ~ 4 days (a signal appeared in InSAR images for T13 by 14th September (Supplementary Fig. 5) and operations ceased by 18th September). The maximum time period is ~ 9 days (injection began on 9th September and T64 showed a full deformation signal on 18th September).

For 2018, the minimum time period of slip is ~ 3 days (a signal appeared in InSAR images for T13 by 15th October (Supplementary Fig. 5) and operations ceased by 18th October). The maximum time period is ~ 12 days (T86 showed no deformation by 8th October and T86 showed a full deformation signal on 20th October).

Results for both SSEs are plotted in Fig. 5.

### Comments on magnitude

The SSE *M*_*w*_ of 5.0 is larger than any seismic event induced by HF in Western Canada to date^68,69^. Studies have linked the maximum magnitude of induced events to fluid volume injected^83,84^, and it is therefore perhaps surprising that such an event occurred on a relatively small pad (i.e. only two wells) and after only around half of the planned HF stages were completed. For 2017, a maximum magnitude of 4.4 would be expected based on^83^, using the total injected volume of 88,473 m^3^ and assuming a shear modulus of 30 GPa, which is a reasonable estimate for 2 km depth in this region based on density and sonic well log data from the region and in comparison to values used in other studies^68^. The observed SSE significantly exceeds this value. However, it does fit within estimates of^84^; for example, a *γ* value of 1.5 × 10^10^ (as defined by^84^) results in a maximum magnitude of 5.7 for the same injected volume. Nevertheless, the magnitude is unusually high for the volume of fluid injected, suggesting that this SSE event may have released some tectonic stress and could therefore be considered 'run-away' or 'triggered'^85^.

### Other injection activities

No other HF treatments were occurring in the immediate vicinity of the slip events during these time periods. However, it is noted that there are two local wastewater disposal wells in the region, located ~ 5 km from the wellhead (to the NW and to the NE, Supplementary Fig. 8). These wells target the Debolt Formation below the Montney Formation and have been operational since 2012 and 2015, with injected volumes of 1,201,573 m^3^ and 432,717 m^3^ as of May 2020, respectively. We cannot rule out the possibility that stress and pore pressure changes from fluid injection at these wells had an effect on the stress state of the slip planes, but due to the significantly shallower depth within the Montney Formation, proximity to the HF wells and timing relationship of the SSEs, HF is interpreted to be the primary triggering mechanism.

## Supplementary Information

Supplementary Information.

## Data Availability

Sentinel-1 data can be downloaded from the NASA Distributed Active Archive Center (DAAC) operated by the Alaska Satellite Facility (ASF, https://search.asf.alaska.edu). Seismicity data are available from the Induced Seismicity Research Project of Natural Resources Canada^71,72,77^. Well data and hydraulic fracturing completions data can be downloaded from the BC Oil and Gas Commission website: https://www.bcogc.ca/energy-professionals/online-systems/.
