# Supplementary material for: InSAR data reveal that the largest hydraulic fracturing-induced earthquake in Canada, to date, is a slow-slip event
Source: Sci Rep. 2022 Feb 7;12:2043. doi: 10.1038/s41598-022-06129-3 (PMC8821710; doi:10.1038/s41598-022-06129-3)
Supplement: Supplementary file 1 — Supplementary Information. [file 41598_2022_6129_MOESM1_ESM.pdf]

Supplementary Materials for

InSAR data reveal that the largest hydraulic fracturing-induced earthquake in Canada, to date, is a slow-slip event

Thomas S. Eyre[1]*, Sergey Samsonov[2], Wanpeng Feng[3], Honn Kao[4,5], David W. Eaton[1]

Correspondence to: thomas.eyre@ucalgary.ca

This PDF file includes:

Supplementary Figs. 1 to 8
Supplementary Tables 1 to 4

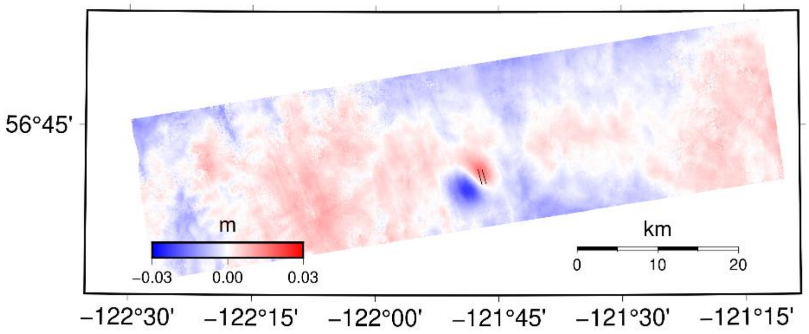

(a) Track 64 (asc), 6 - 18 Sept 2017

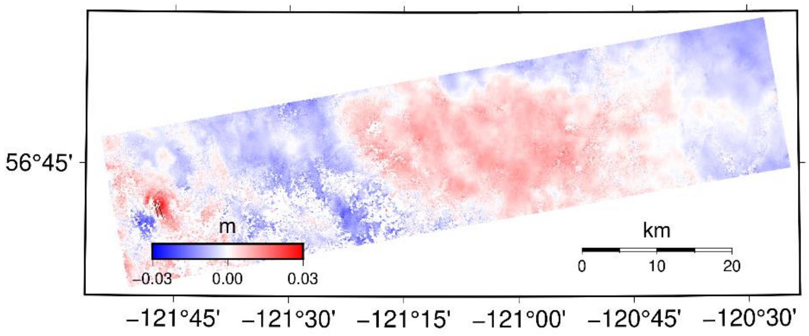

(b) Track 166 (asc), 7 - 19 Sept 2017

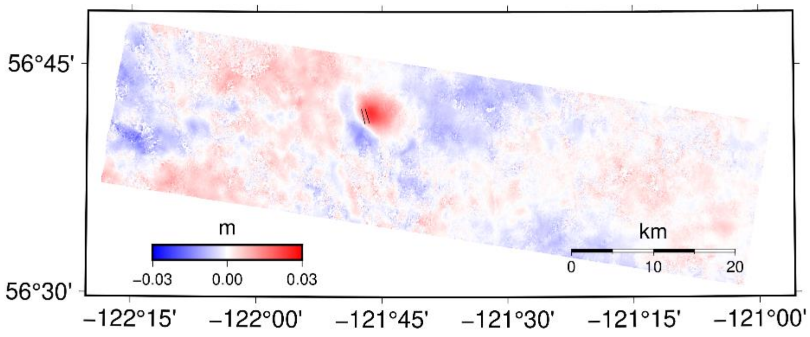

(c) Track 13 (dsc), 9 - 26 Sept 2017

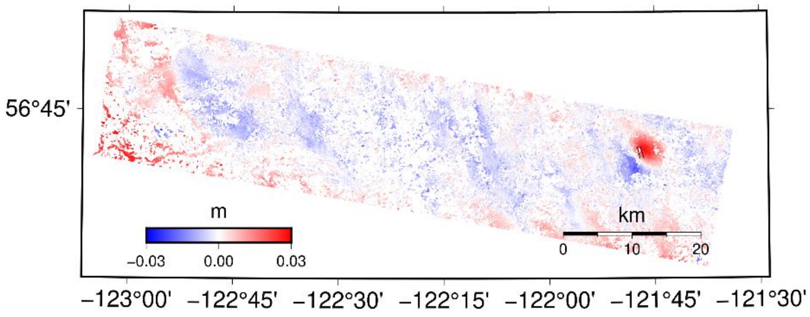

(d) Track 086 (dsc), 7 - 19 Sept 2017

Supplementary Fig. 1.

InSAR data used to model displacements during 2017. Clear positive and negative lobes are seen for each satellite track. HF wells are shown (black lines). Created using (*86*,*87*) (see Acknowledgements for full details).

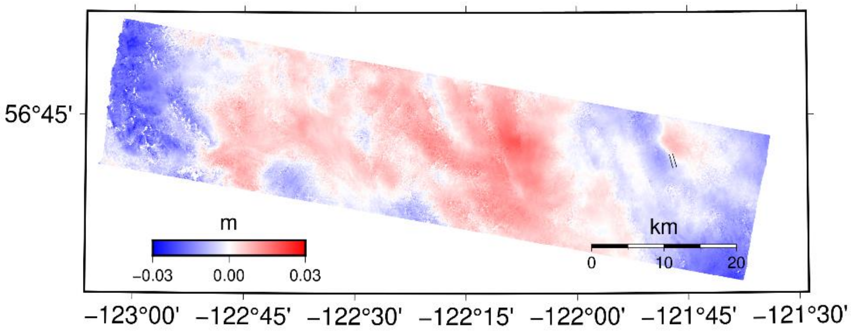

(a) Track 86 (dsc), 8 - 20 Oct 2018

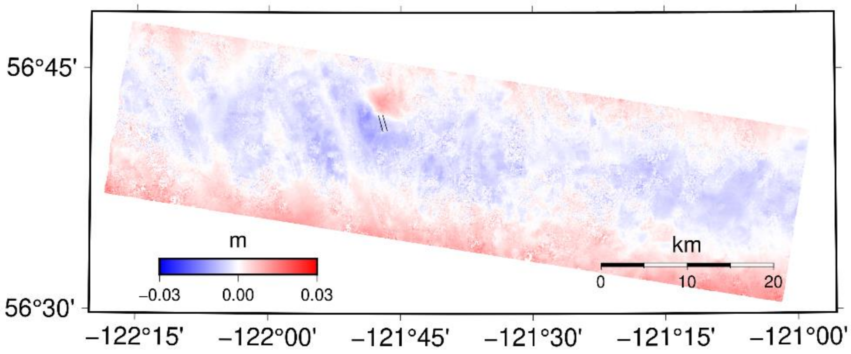

(b) Track 13 (dsc), 3 - 27 Oct 2018

Supplementary Fig. 2.

InSAR data used to model displacements during 2018. Signals are weaker than in 2017 but clear positive and negative lobes can still be seen for each satellite track. HF wells are shown (black lines). Created using (*86,87*) (see Acknowledgements for full details).

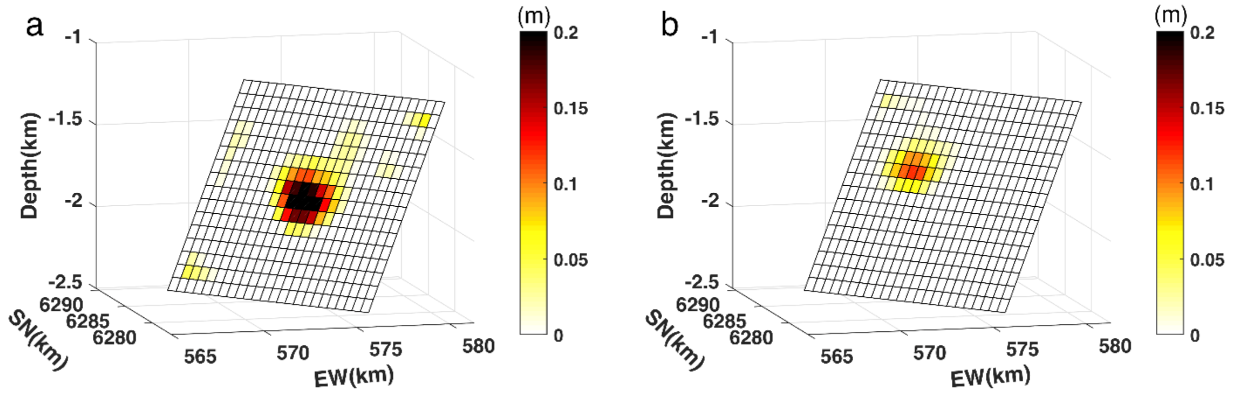

Supplementary Fig. 3.

Results for slip inversion on the 3D modelled fault plane for the events in (A) 2017 and (B) 2018 (note vertical exaggeration). Contoured results are shown with respect to the HF wells in Fig. 2.

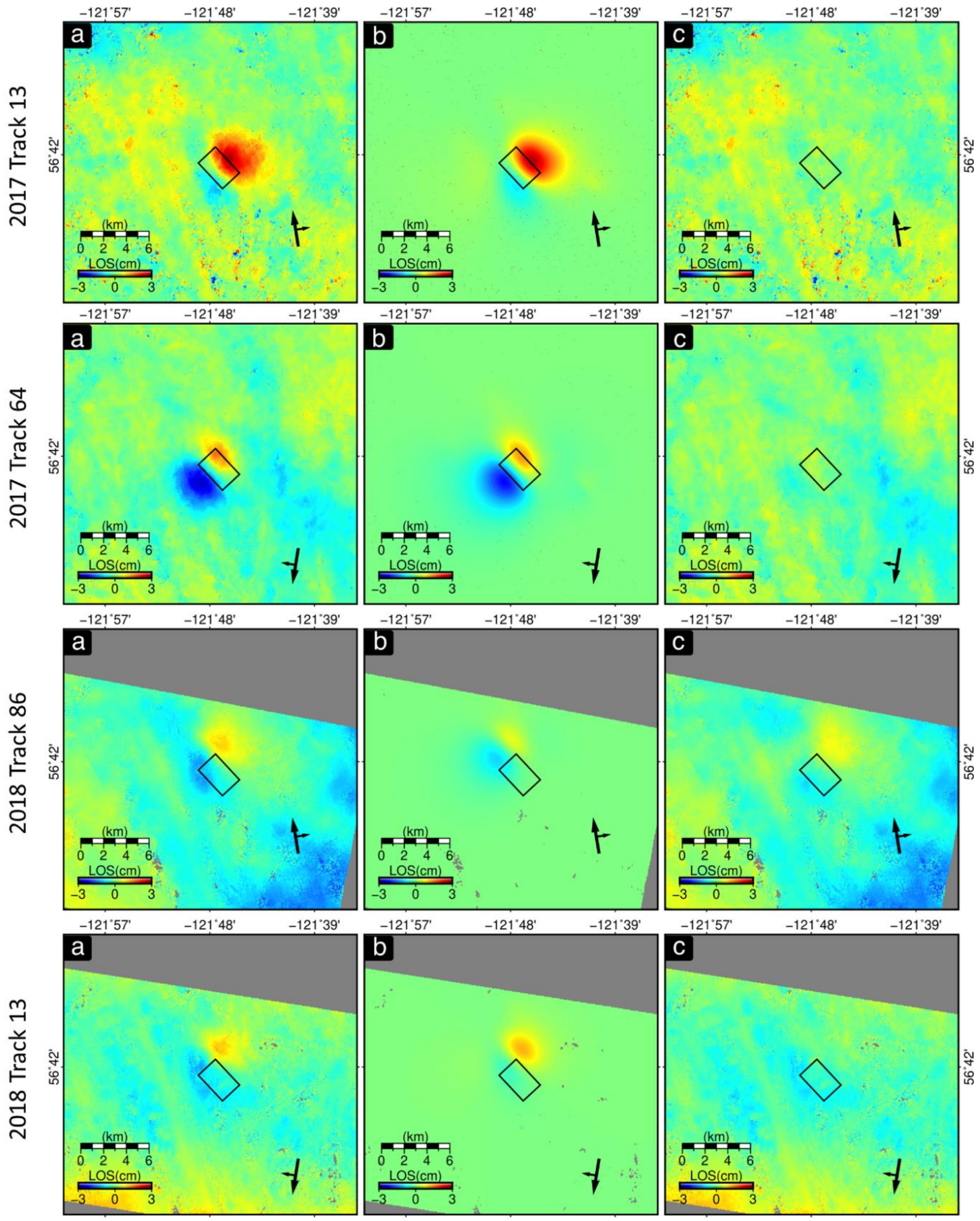

Supplementary Fig. 4.

(a) Data, (b) model, and (c) residual for the events in 2017 and 2018 for different Tracks. LOS = line-of-sight; positive corresponds to mean movements towards the satellite. The model replicates the data very well in each case. Black rectangle = modelled fault plane; large and small arrows denote azimuth and line-of-sight, respectively. Created using (*86,87*) (see Acknowledgements for full details).

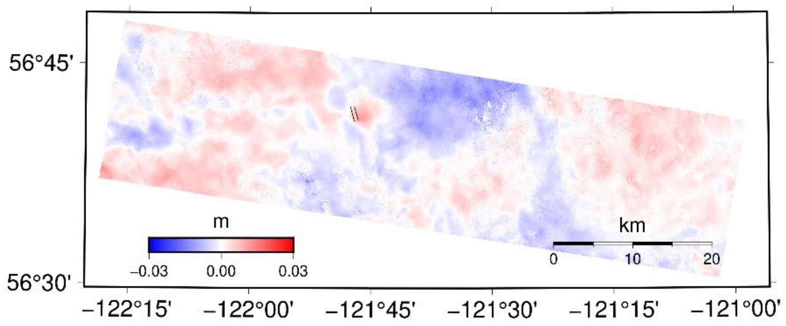

(a) Track 13 (dsc), 9 - 14 Sept 2017

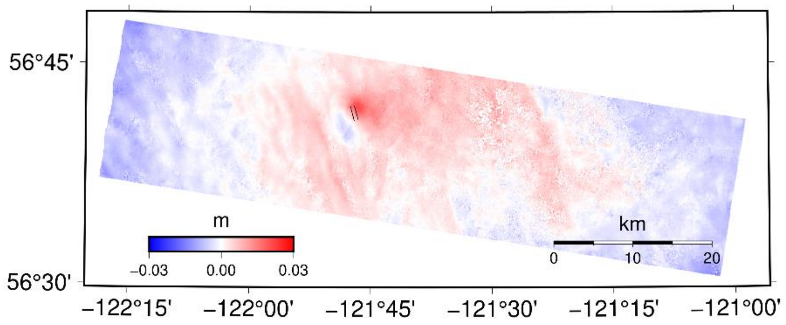

(b) Track 13 (dsc), 14 - 26 Sept 2017

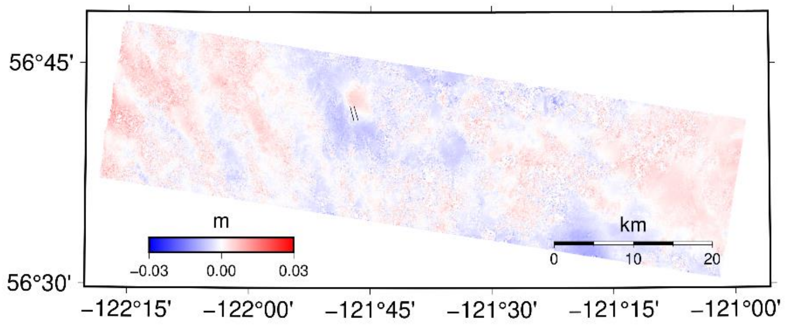

(c) Track 13 (dsc), 3 - 15 Oct 2018

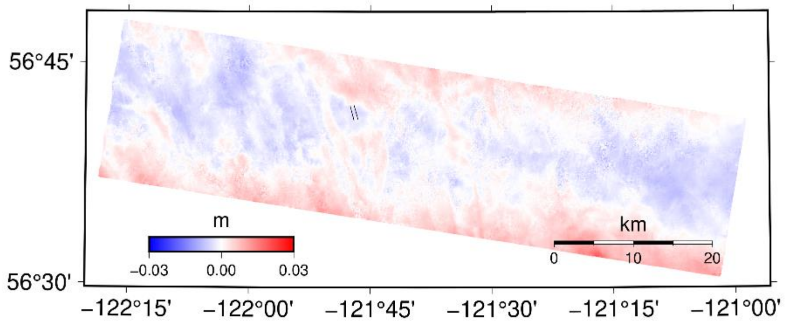

(d) Track 13 (dsc), 15 - 27 Oct 2018

Supplementary Fig. 5.

InSAR data from Track 13 for four different time periods. Positive and negative lobes are seen near the HF wells (black lines), showing deformation in each period that migrates from south to north with completion of the wells. Created using (*86,87*) (see Acknowledgements for full details).

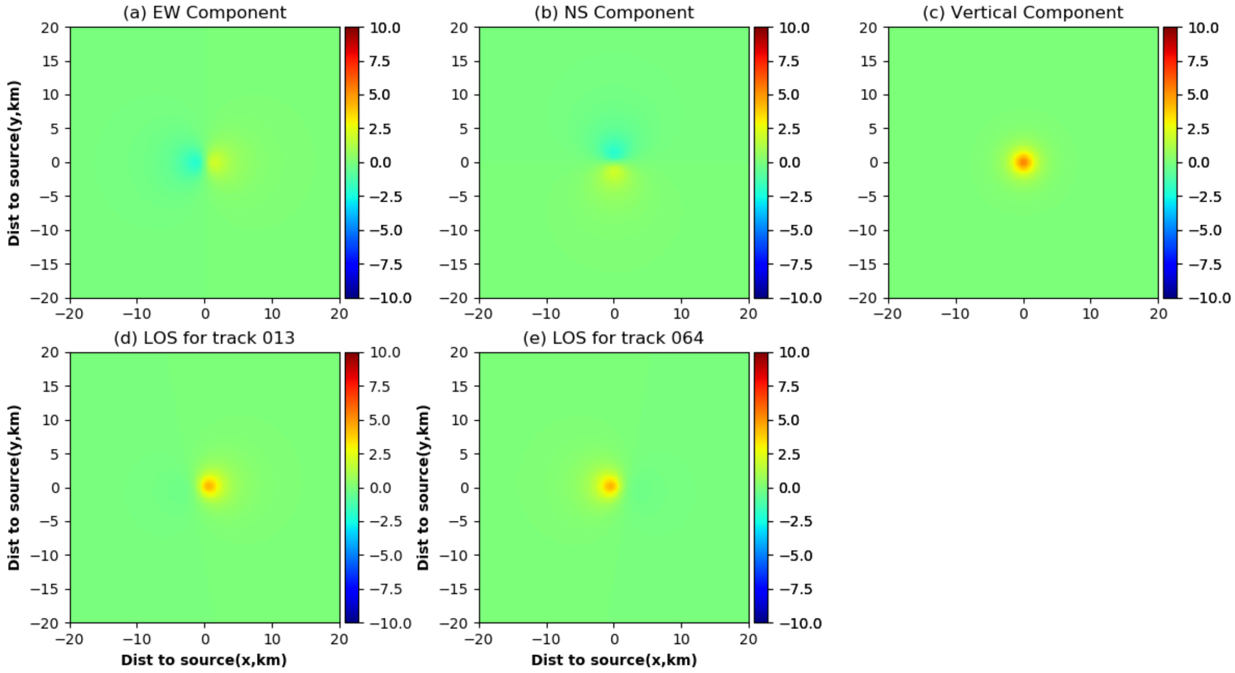

Supplementary Fig. 6.

(a) – (c) Surface deformation (mm) due to fluid injection for each component, using a Mogi source model for the parameters in Table S4. (d) – (e) Synthetic InSAR LOS components generated using the results, with the incidence angles (both 43°) and heading degrees (-170° and -10°) based on Track 013 and Track 064, respectively. Only a few millimeters of uplift are seen in the LOS components, much smaller than the InSAR observations.

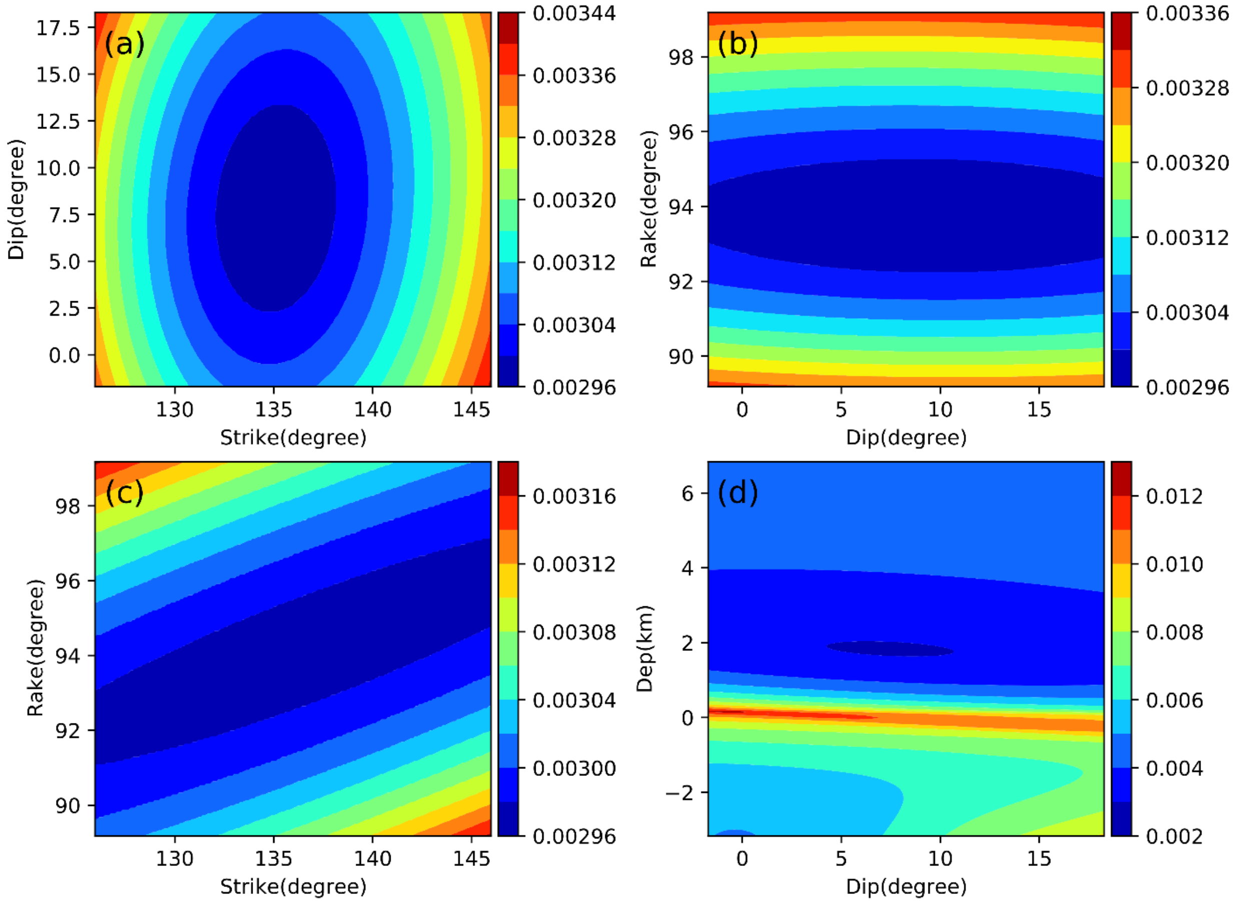

Supplementary Fig. 7.

Trade-off plots of highly nonlinear fault model parameters computed based on a grid-search strategy with centered initial parameters derived from our non-linear global searching. (a), (b), (c) and (d) show the residue distributions for strike-dip, dip-rake, strike-rake and dip-depth, respectively.

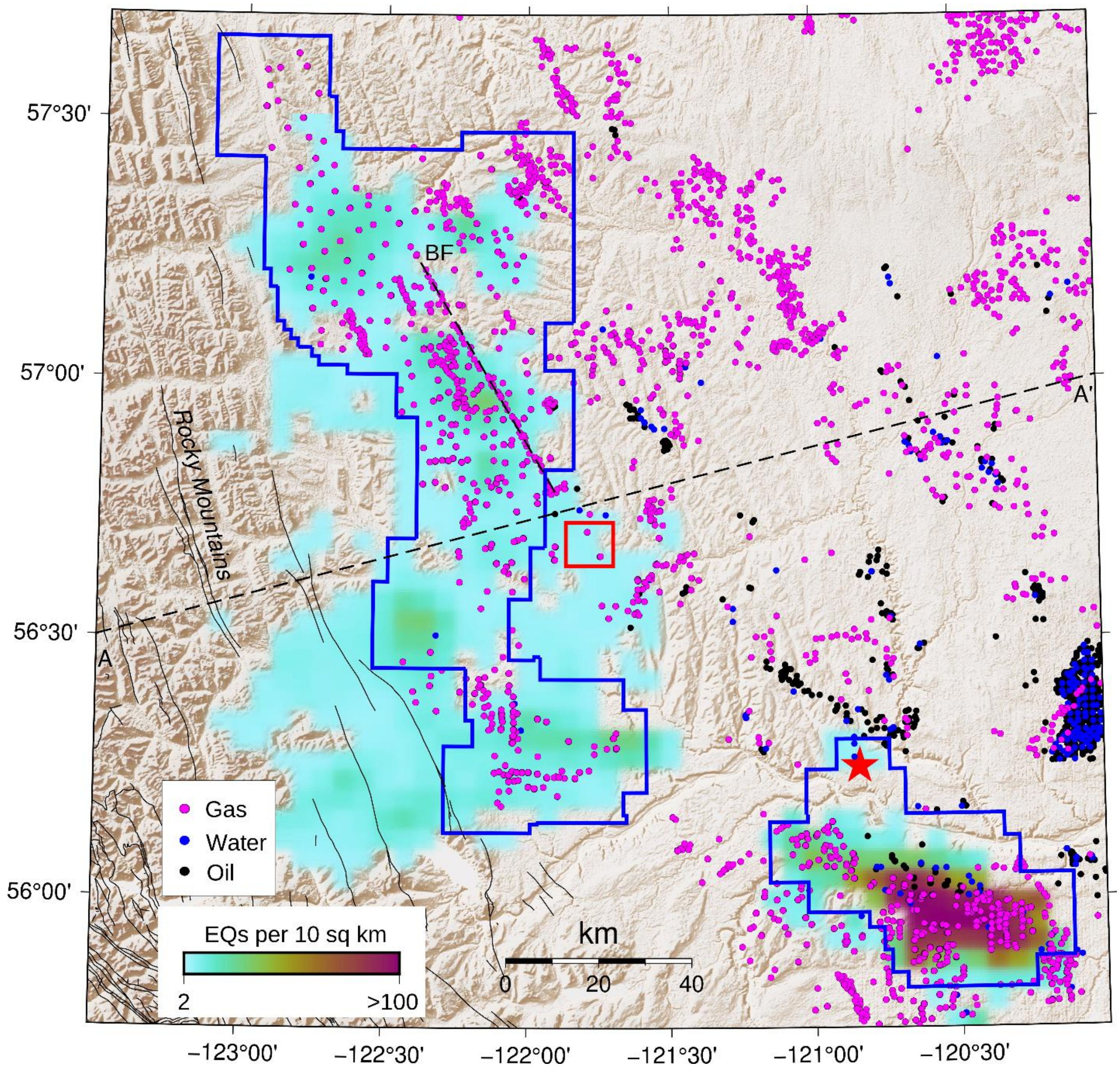

Supplementary Fig. 8.

Similar to Fig. 1 showing the location of different wells in the region classified as active (oil, gas and water injection). Dashed line A-A' shows approximate location of schematic diagram Fig. 3. The Blueberry Fault (BF) is delineated by gas wells targeting the hanging wall and likely continiues through the study area. Created using (*86,87*) (see Acknowledgements for full details).

Date Time	Latitude	Longitude	Depth (km)	Magnitude	Location Algorithm	Velocity Model
12/2/2014 18:49	56.7213	-121.7418	16.3	1.4	dbgenloc	CN01
3/18/2015 15:23	56.68	-121.8573	0.1	1.6	dbgenloc	CN01
6/9/2015 5:32	56.6683	-121.7491	12.2	1.1	dbgenloc	CN01
8/17/2015 8:29	56.694	-121.7753	10	1.5	dbgenloc	CN01
7/23/2016 19:03	56.6569	-121.86	10.8	1.2	dbgenloc	CN01
8/14/2016 2:37	56.7288	-121.8078	8.2	1.5	dbgenloc	CN01
8/27/2016 2:30	56.7106	-121.8163	1	0.8	dbgenloc	CN01
9/9/2016 8:42	56.646	-121.7434	6.9	1.2	dbgenloc	CN01
10/18/2016 1:14	56.6676	-121.7641	0.2	1.5	dbgenloc	CN01
10/21/2016 18:32	56.6409	-121.7808	0.5	1.6	dbgenloc	CN01
11/12/2016 13:11	56.6383	-121.8584	1	1.1	dbgenloc	CN01
2/12/2018 12:55	56.6937	-121.837	7.4	1.7	NLLoc	ALI_NORTHERN_2020
10/22/2018 19:26	56.7208	-121.8523	7.4	1.4	NLLoc	ALI_NORTHERN_2020
1/5/2019 20:28	56.7156	-121.8617	4.1	1.2	NLLoc	ALI_NORTHERN_2020
6/24/2019 4:10	56.6868	-121.7598	2	0.9	NLLoc	ALI_NORTHERN_2020
10/30/2019 2:13	56.6642	-121.8707	4	1.6	NLLoc	ALI_NORTHERN_2020
12/8/2019 5:03	56.6901	-121.8483	4.1	1.3	NLLoc	ALI_NORTHERN_2020
3/20/2020 2:42	56.7296	-121.8357	4	1.2	NLLoc	ALI_NORTHERN_2020
5/9/2020 1:57	56.638	-121.7363	7.5	1.6	NLLoc	ALI_NORTHERN_2020
5/10/2020 3:11	56.6304	-121.7264	7.5	1.4	NLLoc	ALI_NORTHERN_2020
6/14/2020 4:13	56.7118	-121.8486	4	2.5	NLLoc	ALI_NORTHERN_2020

Supplementary Table 1.

Seismic events recorded in the immediate vicinity of the HF wells from 2014-2020 [3–5]. Abbreviations are taken from the referenced sources. No events were recorded during injection periods.

Track	Orbit	Swath	Burst	Images
64	asc	3	3	6, 18 Sept 2017
166	asc	2	6	7, 19 Sept 2017
13	dsc	3	9	2, 14, 26 Sept 2017; 3, 15, 27 Oct 2018
86	dsc	2	5	7, 19 Sept 2017; 8, 20 Oct 2018

Supplementary Table 2.
Sentinel-1 data used in this study.

Modeling period	Track	Perp. baseline, m.	Image 1	Image 2
2017	64	-89	6 Sept 2017	18 Sept 2017
	166	-77	7 Sept 2017	19 Sept 2017
2017	13	-7	2 Sept 2017	26 Sept 2017
	86	32	7 Sept 2017	19 Sept 2017
2018	13	110	3 Oct 2018	27 Oct 2018
2018	86	-69	8 Oct 2018	20 Oct 2018
	13	13	2 Sept 2017	14 Sept 2017
	13	-16	14 Sept 2017	26 Sept 2017
	13	16	3 Oct 2018	15 Oct 2018
	13	94	15 Oct 2018	27 Oct 2018

Supplementary Table 3.
Interferograms used in modelling.

UWI	License #	Date	Stage Latitude	Stage Longitude	Depth B.S.L.	Volume (m^3)	Average Pressure (MPa)	Rate (m^3/s)
200B022K094A1200	31228	9/10/2017	56.68582	-121.775	-1120.75	879.3	49.2	6.8
200B022K094A1200	31228	9/14/2017	56.69025	-121.778	-1120.75	2549.8	51.6	11.4
200B022K094A1200	31228	9/15/2017	56.69075	-121.778	-1120.75	2537.4	51.1	12
200B022K094A1200	31228	9/15/2017	56.69149	-121.778	-1120.75	2488.7	51.1	11.5
200B022K094A1200	31228	9/15/2017	56.69199	-121.779	-1120.75	2440.9	52.2	11.5
200B022K094A1200	31228	9/16/2017	56.69248	-121.779	-1120.75	2557.8	50.6	11.2
200B022K094A1200	31228	9/16/2017	56.69297	-121.779	-1120.75	2444.3	49.3	10.6
200B022K094A1200	31228	9/17/2017	56.69347	-121.779	-1120.75	2586.9	49.7	11.5
200B022K094A1200	31228	9/17/2017	56.69445	-121.78	-1120.75	2542.4	51	11.9
200B022K094A1200	31228	9/10/2017	56.68631	-121.776	-1120.75	2593.5	51	9.6
200B022K094A1200	31228	9/11/2017	56.6868	-121.776	-1120.75	2549.1	52.6	9.8
200B022K094A1200	31228	9/11/2017	56.68729	-121.776	-1120.75	2641.5	51.1	9.7
200B022K094A1200	31228	9/12/2017	56.6878	-121.776	-1120.75	2382.9	52.2	10
200B022K094A1200	31228	9/12/2017	56.68829	-121.777	-1120.75	2485.2	52.4	9.2
200B022K094A1200	31228	9/13/2017	56.68878	-121.777	-1120.75	2684.5	54.8	11.8
200B022K094A1200	31228	9/13/2017	56.68927	-121.777	-1120.75	2552.8	52.6	9.7
200B022K094A1200	31228	9/14/2017	56.68976	-121.777	-1120.75	2433.7	51.2	10
200B022K094A1200	31228	10/13/2018	56.69494	-121.78	-1120.75	3699.6	44.1	12
200B022K094A1200	31228	10/14/2018	56.69567	-121.78	-1120.75	3476.2	43.2	12.1
200B022K094A1200	31228	10/14/2018	56.69617	-121.781	-1120.75	3772.7	44.6	11.7
200B022K094A1200	31228	10/14/2018	56.69665	-121.781	-1120.75	2979.6	42.1	11.7
200B022K094A1200	31228	10/15/2018	56.69714	-121.781	-1120.75	3062.2	46.4	11.3
200B022K094A1200	31228	10/15/2018	56.69762	-121.782	-1120.75	2968.9	44.4	11.5
200B022K094A1200	31228	10/15/2018	56.69811	-121.782	-1120.75	2790.7	43.3	11.9
200B022K094A1200	31228	10/16/2018	56.6986	-121.782	-1120.75	2753	38.7	12
200B022K094A1200	31228	10/16/2018	56.69909	-121.782	-1120.75	2656.1	41.7	12
200B022K094A1200	31228	10/16/2018	56.69958	-121.782	-1120.75	2688.2	42.4	11.9
200B022K094A1200	31228	10/16/2018	56.70006	-121.783	-1120.75	2714.9	41.9	11.9
200B022K094A1200	31228	10/17/2018	56.70055	-121.783	-1120.75	2697.9	41.8	11.9
200B022K094A1200	31228	10/17/2018	56.70103	-121.783	-1120.75	2702.2	42	11.9
200B022K094A1200	31228	10/17/2018	56.70176	-121.784	-1120.75	2690.6	42.1	12
200B022K094A1200	31228	10/18/2018	56.70224	-121.784	-1120.75	2702.8	46.5	11.9
200B023K094A1200	31226	9/9/2017	56.68493	-121.784	-1182.74	1088.1	48.8	10.3
200B023K094A1200	31226	9/14/2017	56.68963	-121.786	-1182.74	3062.6	51	11.6
200B023K094A1200	31226	9/14/2017	56.69013	-121.786	-1182.74	2856.1	50	12
200B023K094A1200	31226	9/15/2017	56.69062	-121.786	-1182.74	2797.5	50.1	11.9
200B023K094A1200	31226	9/15/2017	56.69112	-121.787	-1182.74	2827.9	49.1	12
200B023K094A1200	31226	9/16/2017	56.69162	-121.787	-1182.74	2787.1	47.9	12
200B023K094A1200	31226	9/16/2017	56.69211	-121.787	-1182.74	2978.1	47.9	12
200B023K094A1200	31226	9/17/2017	56.69261	-121.787	-1182.74	2740.5	48.9	12
200B023K094A1200	31226	9/17/2017	56.69335	-121.788	-1182.74	2823.6	48.4	11.9
200B023K094A1200	31226	9/10/2017	56.68542	-121.784	-1182.74	3057.9	51.4	11.6
200B023K094A1200	31226	9/10/2017	56.68591	-121.784	-1182.74	2800.6	52	11.9
200B023K094A1200	31226	9/11/2017	56.6864	-121.785	-1182.74	2732.3	51.6	12
200B023K094A1200	31226	9/11/2017	56.6869	-121.785	-1182.74	2796.3	50.6	11.7
200B023K094A1200	31226	9/12/2017	56.6874	-121.785	-1182.74	2989.3	51.8	11.5
200B023K094A1200	31226	9/12/2017	56.68814	-121.785	-1182.74	2737.8	49.7	11
200B023K094A1200	31226	9/13/2017	56.68864	-121.786	-1182.74	3212.4	49.2	11.2
200B023K094A1200	31226	9/13/2017	56.68914	-121.786	-1182.74	2834.2	51.9	12
200B023K094A1200	31226	10/5/2018	56.69433	-121.788	-1182.74	6742.3	48.9	11.1
200B023K094A1200	31226	10/6/2018	56.69531	-121.789	-1182.74	7560.9	47.6	11.5
200B023K094A1200	31226	10/7/2018	56.69629	-121.789	-1182.74	6397.9	50.1	10.5
200B023K094A1200	31226	10/7/2018	56.69752	-121.79	-1182.74	6514.9	51.2	9.9
200B023K094A1200	31226	10/8/2018	56.6985	-121.79	-1182.74	6675.6	48.8	10
200B023K094A1200	31226	10/9/2018	56.69949	-121.791	-1182.74	6362.6	48.5	11.7
200B023K094A1200	31226	10/9/2018	56.70047	-121.791	-1182.74	7328.3	48.1	10.5
200B023K094A1200	31226	10/10/2018	56.7017	-121.792	-1182.74	6255.1	48.2	9.8

Supplementary Table 4.

HF stage data including timings, locations and injected volumes for the two wells (from BC Oil and Gas Commission). UWI = unique well identifier; the Middle Montney well is 200/B-

022-K 094-A-12/00 and the Lower Montney well is 200/B-023-K 094-A-12/00. Stages were completed from south to north along each well, with 17 completed for each well in September 2017 and the remainder (14 and 8, respectively) in October 2018. Ground surface is 892 m above sea level at the wellpad.
